# Caregiver responses and association with delayed care-seeking in children with uncomplicated and severe malaria

**DOI:** 10.1186/s12936-018-2630-9

**Published:** 2018-12-18

**Authors:** Arthur Mpimbaza, Anne Katahoire, Philip J. Rosenthal, Charles Karamagi, Grace Ndeezi

**Affiliations:** 1 Child Health and Development Centre, Makerere University, College of Health Sciences, Kampala, Uganda; 2 Department of Medicine, University of California, San Francisco, USA; 3 Department of Pediatrics and Child Health, Makerere University, College of Health Sciences, Kampala, Uganda; 4 Clinical Epidemiology Unit, Department of Medicine, Makerere University, College of Health Sciences, Kampala, Uganda

**Keywords:** Caregiver, Responses, Delayed care-seeking, Malaria

## Abstract

**Background:**

Gaps remain in understanding the role of caregiver responses on time to seek appropriate care. The objective of this study was to describe caregiver responses to illness and the impact of these responses on time to seek appropriate care among children with malaria.

**Methods:**

A case–control study of 325 children with severe (cases) and 325 children with uncomplicated (controls) malaria was conducted in Jinja, Uganda. Caregivers’ responses to their children’s illnesses and time to seek appropriate care were documented. Responses included staying at home, seeking care at drug shops, and seeking care at public health facilities classified into two types: (1) health facilities where caregiver initially sought care before enrollment, and (2) health facilities where children were provided appropriate care and enrolled in the study. Weighted Cox regression was used to determine risk factors for delays in time to seek appropriate care within 24 h of illness onset.

**Results:**

Children staying home on self-medication was the most common initial response to illness among caregivers of controls (57.5%) and cases (42.4%, p < 0.001), followed by staying at home without medication (25.2%) and seeking care at drug shops (32.0%) for caregivers of controls and cases, respectively. Seeking care at drug shops was more common among caregivers of cases than of controls (32.0% vs. 12.3%; p < 0.001). However, compared to public health facilities, drug shops offered sub-optimal services with children less likely to have been examined (50.0% vs. 82.9%; p < 0.001) or referred to another facility (12.5% vs. 61.4%; p < 0.001). Upon adjustment for known risk factors for delay, initially seeking care at a drug shop (HR 0.37, p = 0.036) was associated with delay in seeking care at a health facility where appropriate care was provided. In contrast, those initially seeking care at public health facility before enrollment were more likely to subsequently seek care at another public health facility where appropriate care was provided (HR 5.55, p < 0.001).

**Conclusion:**

Caregivers should be educated on the importance of promptly seeking care at a health facility where appropriate care can be provided. The role of drug shops in providing appropriate care to children with malaria needs to be reviewed.

**Electronic supplementary material:**

The online version of this article (10.1186/s12936-018-2630-9) contains supplementary material, which is available to authorized users.

## Background

The World Health Organization (WHO) emphasizes prompt (within 24 h of fever onset) treatment with effective anti-malarials as the main strategy for prevention of progression from uncomplicated to severe malaria [1, 2]. Concerted efforts by governments, international organizations, and partner institutions have resulted in increased availability of high quality and affordable artemisinin-based combination therapy (ACT) in both the public and private sectors of health [3–6]. Increased availability of ACT has likely contributed to reductions in malaria morbidity and mortality in sub-Saharan Africa [7–9]. However, despite progress, a large number of African children continue to die of malaria, partly due to delays in seeking appropriate care [10]; defined as prompt diagnosis by microscopy or RDTs for suspected malaria cases followed by treatment with ACT, if uncomplicated malaria is diagnosed, or parenteral artesunate and supportive care if severe malaria is diagnosed. For children with severe malaria, if appropriate care cannot be provided at the facility the child should be referred to a facility where care can be provided.

The proportion of African children with confirmed malaria receiving ACT has been reported to vary greatly, ranging from a low of 0.6% in Somalia to a high of 70.2% in Uganda [11]. Of concern, the proportion of children with confirmed malaria promptly receiving ACT is unacceptably low, ranging from 2 to 22% in 17 high burden countries in Africa [6]. Low provision of appropriate treatment for uncomplicated malaria is partly explained by preferential attention given to health system factors at the expense of focus on individual actions [12]. Time taken to seek appropriate care by caregivers of sick children is influenced by two important decisions: when to respond to illness and where to seek care [13]. These decisions determine the time to seek appropriate care. In sub-Saharan Africa, responses to seek care outside home are often delayed, and when a response is made, this response is often to seek care at a drug shop, rather than at a qualified health facility [14, 15].

Appreciation of the significance of caregiver responses to illness as determinants of promptly seeking appropriate care is increasing. However, despite their importance, the impact of these responses on time to seek appropriate care remains largely unexplored. As part of a case control study of risk factors for severe malaria, presented are caregivers’ responses to illness in children with uncomplicated and severe malaria. Predictors of different responses and impact of initial responses on time to seek appropriate care were also studied.

## Methods

### Study design

**Fig. 1:**
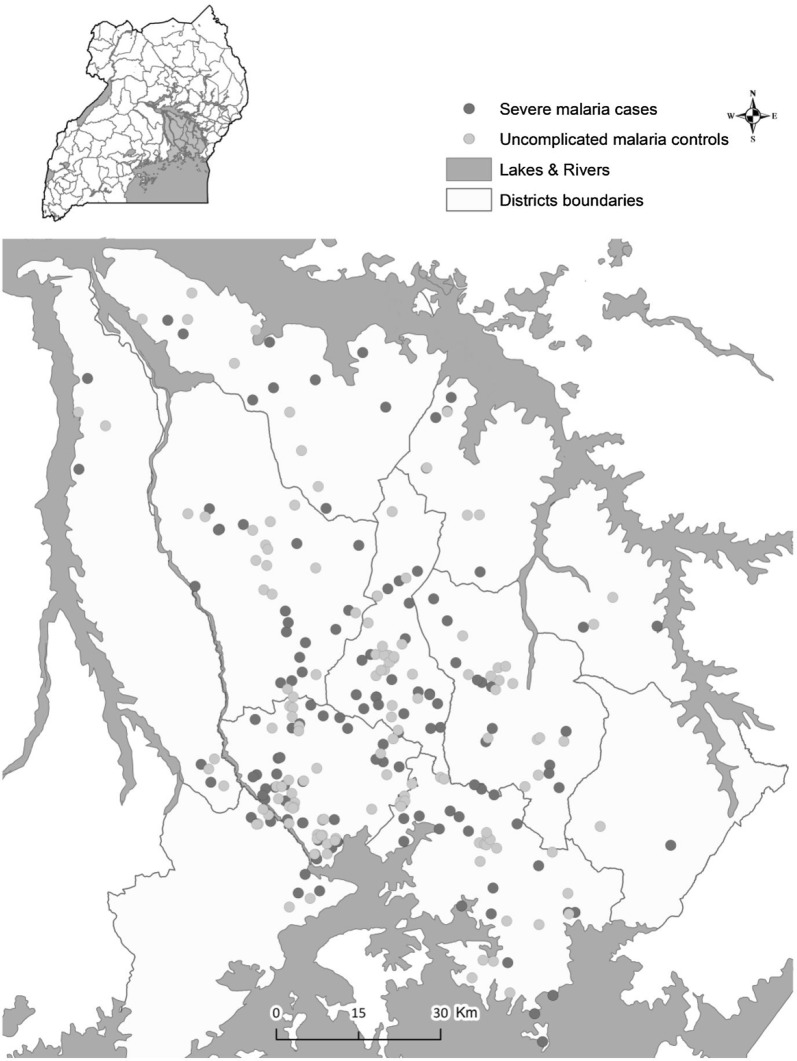
Study site. Map demonstrating locations of residence’s for both severe malaria cases and uncomplicated malaria cases within the catchment area of Jinja Hospital

A matched case–control study was conducted to identify determinants of severe malaria in Ugandan children, as recently described [16]. In brief, 325 severe malaria cases and 325 uncomplicated malaria controls were enrolled. Severe malaria cases were enrolled at the Children’s Ward, Jinja Regional Referral Hospital (JRRH). Uncomplicated malaria controls were enrolled at a level III or higher public health facility, geographically matched (level of sub-county) to the residence of an age-matched case. Additionally, controls were matched to cases by calendar time (enrollment within 1 month of case). The latter two matching criteria were intended to result in selection of controls with an exposure distribution identical to that of the population that gave rise to the cases. Information concerning the child’s caregiver, head of household (relation to child, age, education level, employment status), and house characteristics and possessions were also documented and used to construct a wealth index for each child. GPS coordinates of participants’ homes were captured and used to determine distances between these homes and the nearest public health facility (Fig. 1). Information on caregivers’ responses to illness was systematically re-constructed into an itinerary detailing events that took place throughout the child’s illness reflecting progression of illness against response and actions taken from illness onset to the date and time when the child was enrolled. The itinerary was a comprehensive account of each caregiver’s pathway to seeking appropriate care.

### Responses

Caregiver responses to illness were categorized as staying at home or care seeking, defined as any care sought outside the home. If the caregiver provided the child medicine without having the child reviewed by a care-provider, this was referred to as staying home on self-medication. Public health facilities where caregiver sought care were categorized into two types, facilities where caregivers initially sought care before presenting to the facility where appropriate care was provided, and facilities where caregivers sought care leading to provision of appropriate care and enrollment in the study. Care seeking at private health facilities and non-biomedical sources of care were also captured. To distinguish drug shops from private clinics, drug shops were defined as facilities where caregivers could buy medicine without the need for the patient to be present. Private clinics were defined as facilities that offered care, with the patient required to pay a fee to be seen by a health worker and facilities for administering intravenous drugs.

### Itinerary of events data

Each caregiver’s itinerary started on the date of illness onset and ended on the date when the child arrived at a public health facility where the child was provided appropriate care and enrolled in the study. To determine the date of illness onset, caregivers were asked to recall the date when the child was last well. Using that date as a reference point, interviewers probed the caregiver about the subsequent daily state of the child’s health. The day when the caregiver reported that the child’s health had deviated from normal was documented as the start date of illness. Starting from that date, caregivers were asked a standard set of questions (Additional file 1) concerning each day of illness, regarding observed symptoms including fever, signs, and response. If the response was care-seeking, caregivers were asked to specify where they sought care, what intervention was provided, and who made care decisions. Additional information related to health services offered by care-providers before enrollment was captured based on caregiver reports. Specifically, we asked if the provider: (1) examined the child, confirmed by having touched the child, and (2) performed laboratory tests for malaria testing and haemoglobin estimation. Provision of anti-malarials to children was also queried. Efforts were made to validate caregiver reports based on description of medicines, and available prescription notes. An event constituted reported symptoms, response, and intervention(s) given, and was defined by the response. Each event represented a step in the caregiver’s itinerary, with subsequent days representing distinct steps in the caregiver’s itinerary, even if the response did not change. Different responses on the same day were captured as distinct events.

### Analysis

Data were entered using Microsoft Access (Microsoft Corporation) and analyzed using STATA (version 14; STATA Corp., College Station, TX, USA). Caregiver responses were grouped as follows: (1) stayed home; not on medication, (2) stayed home on medication, (3) sought care at a drug shop, (4) sought care at a non-biomedical provider, (5) sought care at a private clinic, and (6) sought care at public health facilities ranging from level II to hospitals. For analysis of association between initial response types and time to seek care, care-seeking at private health facilities and non-biomedical providers (< 1% of responses) were collapsed under the categories drug shops and stayed home not on medication, respectively. Time (in days) to seek appropriate care was calculated based on the duration between time when fever was first noted and time of arrival at the enrolling health facility. As cases and controls were matched, the Wilcoxon matched pair signed-rank and the Chi square tests were used for testing the significance of differences between continuous and categorical data in cases and controls, respectively. Caregiver actions were compared based on the initial response by the caregiver and the sum of responses throughout the itinerary of events. Additionally, frequencies of different responses at different time points (equivalent to steps in itineraries) on caregivers’ pathways to care were compared. Health services offered by providers’ were presented as proportion of children who receiving a specified service among those who sought care at the provider. This analysis was limited to children who had severe malaria, as they were more likely to have sought care at an initial facility. Risk factors for care seeking at drug shops as the initial response to illness were determined using logistic regression with a dichotomous outcome (seeking care at a drug shop vs. others). As this outcome was not the basis for the case–control study, weighted logistic regression analysis was performed to account for the biased representation of the outcome amongst cases, as has been described by others [17]. The population incidence of cases was estimated to be 2000 cases per 100,000 people per year (0.02) based on the World Health Organization Uganda malaria country profile [18]. A logistic regression backward stepwise approach (p = 0.2) was used to identify variables for inclusion in the final logistic regression model. Excluded variables that contributed to model fitness and interpretation were retained in the model. Variables were also excluded for collinearity.

Life tables were used to demonstrate patterns of seeking appropriate care at different intervals by caregivers of cases and controls. Weighted Kaplan–Meier survival curves and the Cox regression model were used to determine the un-adjusted and adjusted association between initial caregiver responses and time taken to seek appropriate care within the first 24 h of illness onset, respectively.

## Results

### Care-seeking characteristics

**Fig. 2:**
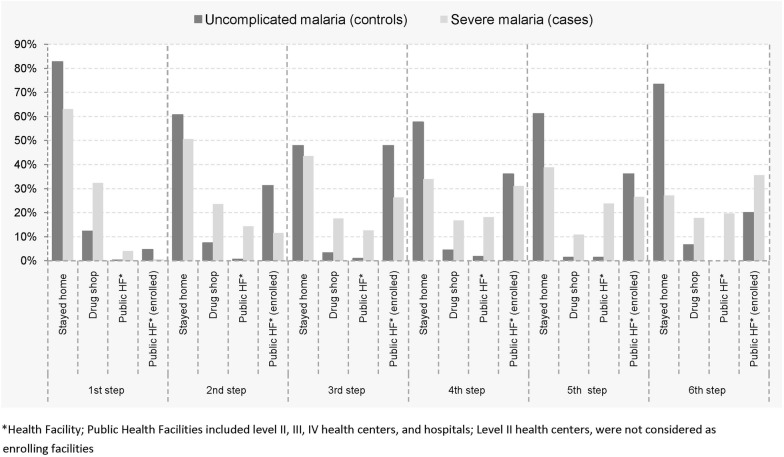
Frequency of responses at different points in caregiver pathways to care. The analysis was limited to the first six steps of caregiver itineraries, when most (> 80%) caregivers had sought appropriate care. The category stayed home included those on medication and not on medication

Caregivers of children with severe malaria (cases) took longer to seek appropriate care (median 2.4 vs. 1.7 days; p < 0.001) and had more responses (median 4.0 vs. 3.0; p < 0.001) compared to caregivers of children with uncomplicated malaria (controls, Table 1). Considering initial responses, overall, staying home on self-medication (49.5%) was the most common response followed by staying at home not on medication (22.9%) and care-seeking at a drug shop (22.1%). Among controls, staying home on self-medication (57.3%) was the most common response followed by staying home not on medication (25.2%) and care-seeking at a drug shop (12.4%). For cases, staying home on self-medication (42.1%) was the most common response followed by care-seeking at a drug shop (32.0%) and staying at home not on medication (20.6%). By comparison, staying home on self-medication (57.2% vs. 42.1%; p < 0.001) was more common among caregivers of controls compared to those of cases. Additionally, initially care-seeking at public health facilities (4.6% vs. 0.6%, p < 0.001) where children were provided appropriate care and enrolled, was more common among controls than cases. In contrast, initially care seeking at drug shops (32.0% vs. 12.3%; p = 0.001) and care-seeking at a public health (4.0% vs. 0%, p < 0.001) before enrollment was more common among caregivers of cases than controls. When the sum of responses were considered, caregivers of controls were more likely to have stayed at home (on medication; 42.4% vs. 26.0%), compared to caregivers of cases. In contrast, caregivers of cases were more likely to have sought care at drug shops (18.3% vs. 6.8%, p < 0.001) and at a public health facility (12.3% vs. 0.7%, p < 0.001; Table 1), compared to caregivers of controls. Consideration of caregiver pathways to care showed that differences in frequencies of occurrence of staying at home (more frequent among controls) or seeking care at drug shops (more frequent among cases) were consistent throughout pathways (Fig. 2).

**Table 1:** Care-seeking characteristics of caregivers responding to illness of their children with uncomplicated and severe malaria

Variable	Caregivers of children with uncomplicated malaria	Caregivers of children with severe malaria	p value
Total number of caregivers, N	325	325	
Median time in days taken to seek appropriate treatment; (IQR)	1.70 (0.91–2.87)	2.45 (1.62–3.81)	< 0.001
Median number of actions taken before seeking appropriate treatment; (IQR)	3 (2–4)	4 (3–6)	< 0.001
Stayed home not on medication	82 (25.2%)	67 (20.6%)	0.169
Stayed home on medication	186 (57.2%)	137 (42.1%)	< 0.001
Drug shop	40 (12.3%)	104 (32.0%)	< 0.001
Non-biomedical provider	2 (0.62) %	1 (0.3%)	0.563
Private clinic	0	1 (0.3%)	*
Initial response-type by caregivers, n (%)^a^			
Health centre II	0	5 ((1.5%)	0.020
Initial health centre III/IV	0	6 (1.8%)	0.014
Enrolling health centre III/IV	11 (3.3%)	0	< 0.001
Initial public hospital	0	2 (0.6%)	0.157
Enrolling public hospital	4 (1.2%)	2 (0.6%)	0.414
Total number of responses taken by caregivers, N	1172	1551	
Stayed home not on medication	254 (21.7%)	276 (17.8%)	0.010
Stayed home on medication	497 (42.4%)	403 (26.0%)	< 0.001
Drug shop	80 (6.8%)	284 (18.3%)	< 0.001
Non-biomedical provider	8 (0.7%)	7 (0.5%)	0.478
Total number of types of responses by caregivers, n (%)^d^			
Private clinic	0	47 (3.0%)	< 0.001
Health centre II	0	19 (1.2%)	< 0.001
Initial health centre III/IV^b^	7 (0.6%)	96 (6.2%)	< 0.001
Enrolling health centre III/IV	258 (22.0%)	0	NA
Initial public hospital^b^	1 (0.1%)	94 (6.1%)	< 0.001
Enrolling public hospital^c^	67 (5.7%)	325 (21.0%)	NA

*NA* not applicable

* Insufficient numbers

^a^Column frequency: numerator: number of type of initial response; denominator: number of caregivers of cases or controls

^b^Initial public facilities where caregiver sought care before enrollment

^c^Enrolling public facilities (level III and above) where caregivers sought care and were provided appropriate care and had their children enrolled in the study

^d^Column frequency: numerator: total number of responses taken by caregivers; denominator: total number of actions taken by caregivers of cases or controls

### Health services provided to children at facilities before enrolment with severe malaria

Compared to private clinics or public health facilities, drug shops provided sub-optimal health service (Table 2). Considering physical examination, drug shops examined fewer children compared to those seen at private clinics (50% vs. 78%, p < 0.001) or all public health facilities (50.0 vs. 82.9%, p < 0.001; Table 2). Malaria testing rates were low across all facilities, but highest at public hospitals (43.5%), private clinics (42.0%), and level IV public facilities (41.3%), and lowest at drug shops (8.9%, p < 0.001 compared to all other sites). Hemoglobin testing was not done at drug shops, but was provided at public facilities (26.8%; range 5.9% at health centre II to 43.5% at public hospitals) and private clinics (28.0%). Provision of anti-malarials to presenting children was highest at drug shops and level II public facilities and lowest at public hospitals (Table 2). Consider patient referral, drug shops were less likely to refer children compared to all public hospitals (12.5% vs. 61.4%, p < 0.001).

**Table 2:** Health services provided to children with severe malaria before enrolment

Facility	Number of children examined by provider n/N (%)	Number of children offered a malaria test n/N (%)	Number of children offered a haemoglobin test n/N (%)	Number of children provided an anti-malarial n/N (%)	Number of children who were referred to another facility n/N (%)
Drug shop	140/280 (50.0%)	25/280 (8.9%)	0/280 (0)	176/280 (62.9%)	35/280 (12.5%)
Private clinic	39/50 (78.0%)	21/50 (42.0%)	14/50 (28.0%)	14/50 (26.9%)	31/50 (62.0%)
Public health centre II	13/17 (76.5%)	5/17 (29.4%)	1/17 (5.9%)	8/17 (47.1%)	9/17 (52.9%)
Public health centre III	25/33 (75.8%)	12/33 (36.4%)	2/33 (6.1%)	8/33 (24.2%)	21/33 (63.6%)
Public health centre IV	54/63 (85.7%)	26/63 (41.3%)	12/63 (19.0%)	21/63 (33.3%)	31/63 (49.2%)
Public hospital	78/92 (84.8%)	40/92 (43.5%)	40/92 (43.5%)	14/92 (15.2%)	66/92 (61.7%)
All public facilities^a^	170/205 (82.9%)	83/205 (40.5%)	35/205 (26.8%)	51/205 (24.8%)	127/205 (62.0%)

^a^All public facilities merged: Level II, III, IV and hospitals

### Factors associated with care-seeking at a drug shop as the initial response to illness

With unadjusted analysis, increasing age of the head of the household (OR 0.94, p < 0.001) was protective against initially care seeking at a drug shop. Upon adjustment, employed caregivers were two times more likely to have sought care at a drug shop compared to those who were not employed (OR 2.33, p = 0.017). Older caretakers (OR 0.95, p = 0.043) and first decision by the child’s mother (OR 0.38, p = 0.026) were protective against care seeking at a drug shop. When the adjusted analysis was limited to caregivers with available GPS data, longer distance to the nearest public health facility (level III and above; OR 1.07; p = 0.397) was not a risk factor for care seeking at a drug shop as the initial response (Table 3).

**Table 3:** Unadjusted and adjusted analysis for determinants of seeking care at a drug shop as the initial response to illness

Variables	All children (N = 325 pairs)	
	Univariate	Multivariable
	OR (95% CI); p-value	OR (95% CI); p-value
Mother took decision on first day	0.76 (0.35, 1.61); 0.478	0.38 (0.16, 0.89); 0.026
Child		
Age in years	0.87 (0.67, 1.13); 0.319	
Female	0.69 (0.36, 1.32); 0.273	
Danger symptoms on day 1	1.26 (0.37, 4.23); 0.704	
Exclusively breast feed for 6 months	1.59 (0.76, 3.33); 0.211	
Sleeps under a net	2.00 (0.71, 5.61); 0.188	2.01 (0.69, 5.80); 0.195
Caregiver		
Age in years	0.97 (0.93, 1.01); 0.146	0.95 (0.91, 0.99); 0.043
Mother of child	0.77 (0.32, 1.86); 0.569	
Post primary education	0.49 (0.06, 3.56); 0.482	
Employed	1.64 (0.85, 3.18); 0.137	2.33 (1.16, 4.69); 0.017
Polygamous relationship	0.93 (0.42, 2.03); 0.859	
Head of home		
Age in years	0.96 (0.93, 0.98); 0.007	
Post primary education	1.46 (0.72, 2.97); 0.288	
Employed	1.54 (0.37, 0.58); 0.379	
≥ 3 children under 5 years of age in the home, n (%)	1.39 (0.59, 3.24); 0.751	
Socioeconomic position		
1 (lowest)	Referent	
2	0.68 (0.25, 1.85); 0.461	
3	1.36 (0.58, 3.21); 0.475	
4 (highest)	0.90 (0.35, 2.31); 0.840	

### Time to seeking appropriate care

Considering the entire itineraries, caregivers of controls were more likely to have sought appropriate care earlier compared to caregivers of cases (Table 4, log rank test 0.001). Differences in time to seek appropriate care were most significant during the first 24 h, when 30.4% of caregivers of controls (hazard 0.35, 95% CI 0.28, 0.42), but only 11.6% of caregivers of cases (hazard 0.12, 95% CI 0.08, 0.16) had sought appropriate care. By 48 h after illness onset, 60.0% of caregivers of controls had sought appropriate care compared to only 35.5% of caregivers of cases (hazards 0.53, 95% CI 0.43, 0.64 and 0.30, 95% CI 0.24, 0.37, respectively). Thereafter, probabilities of seeking appropriate care were similar.

**Table 4:** Life table: cumulative probability, and hazard of seeking appropriate care at different time intervals for caregivers of children with uncomplicated malaria and severe malaria

Interval in days	Caregivers of children with uncomplicated malaria, N = 325				Caregivers of children with severe malaria, N = 325			
	Number of caregivers responding to illness	Number of children who accessed appropriate care	Cumulative probability of accessing appropriate care	Hazard (95%)	Number of caregivers responding to illness	Number of children who accessed appropriate care	Cumulative probability of accessing appropriate care	Hazard (95%)
0 to < 1	325	99	0.304	0.35 (0.28, 0.42)	325	38	0.116	0.12 (0.08, 0.16)
1 to < 2	226	96	0.600	0.53 (0.43, 0.64)	287	77	0.353	0.30 (0.24, 0.37)
2 to < 3	130	55	0.769	0.53 (0.40, 0.67)	210	90	0.630	0.54 (0.43, 0.65)
3 to < 4	75	26	0.849	0.41 (0.26, 0.57)	120	46	0.772	0.47 (0.34, 0.60)
4 to < 5	49	15	0.895	0.36 (0.18, 0.54)	74	23	0.843	0.36 (0.22, 0.51)
5 to < 6	34	9	0.923	0.30 (0.10, 0.50)	51	19	0.901	0.45 (0.25, 0.65)
6 to < 7	25	6	0.941	0.27 (0.05, 0.48)	32	14	0.944	0.56 (0.27, 0.84)
7 to < 8	19	8	0.966	0.53 (0.17, 0.88)	18	10	0.975	0.76 (0.32, 1.20)
8 to < 9	11	6	0.984	0.75 (0.19, 1.30)	8	5	0.990	0.90 (0.19, 1.61)
9 to < 10	5	3	0.993	0.85 (0.00, 1.73)	3	1	0.993	0.40 (0.00, 1.16)
11 to < 12	2	2	1.00	2.00 (2.00, 2.00)	2	1	0.996	0.66 (0.00, 1.89)
12 to < 13					1	1	1	2.00 (2.00, 2.00)

### Impact of care-seeking responses on time to seek appropriate care

**Fig. 3:**
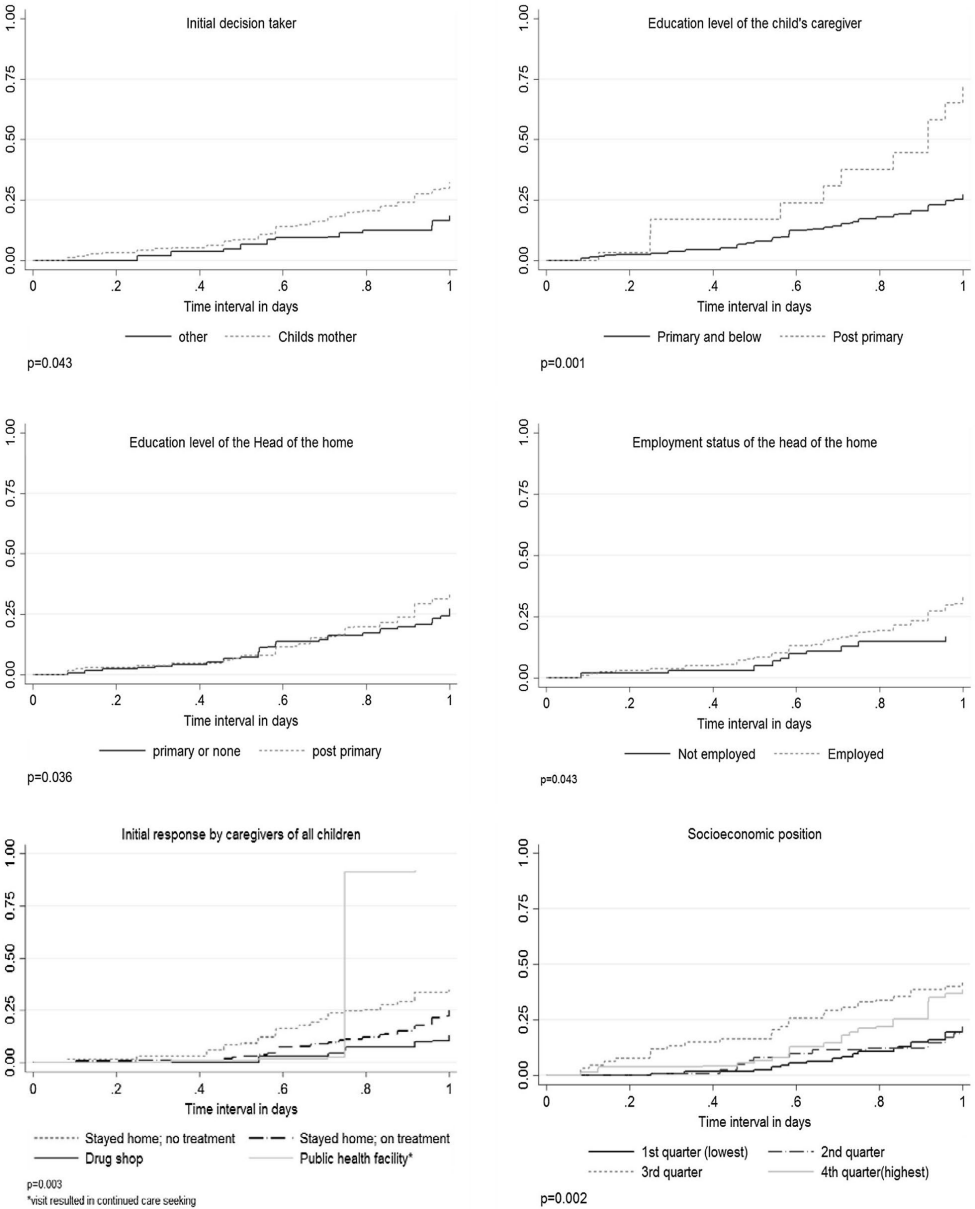
Kaplan Meier Curves for probability of caregivers seeking appropriate care based on different explanatory variables

Using Kaplan–Meier survival curves, initial responses (Cox test p = 0.003), socio-economic position (Cox test p = 0.002), and having a caregiver with post primary education (Coxtest p = 0.001) were significantly associated with prompt seeking of appropriate care within the first 24 h of fever onset (Fig. 3). Upon adjustment, caregivers who initially sought care at a drug shop (HR 0.37; 95% CI 0.14, 0.93, p = 0.036) were nearly three times more likely to delay in seeking appropriate care within the first 24 h. These findings contrast with those of caregivers who initially sought care at a public health facility (HR 5.55; 95% 2.12, 14.5, p < 0.001) prior to enrollment in the first 24 h, who were more likely to have sought care at a public health facility where appropriate care was provided within the same interval (Table 5). In the final model, compared to those with lower levels of education, caregivers with post-primary education were more likely to have sought care early (Table 5).

**Table 5:** Cox regression models for the association between initial responses to illness and seeking appropriate care within 24 h of illness onset

Variable	Hazard Ratio; (95% CI)	p-value
Initial response		
Stayed home; not on medication	Referent	Referent
Stayed home; on medication	0.68; (0.40, 1.17)	0.168
Went to a drug shop	0.37; (0.14, 0.93)	0.036
Initial public hospital (not enrolled)	5.55; (2.12, 14.5)	0.000
Care taker post primary education	2.65; (1.37, 5.76)	0.007
Mother took decision on first day	1.53; (0.74, 3.13)	0.243
Head of household employed	1.62; (0.73, 4.08)	0.237
Socioeconomic position		
1st (lowest)	Referent	Referent
2nd	1.07; (0.51, 2.24)	0.853
3rd	1.61; (0.78, 3.35)	0.196
4th (highest)	1.35; (0.65, 2.78)	0.411

Test of proportional hazards assumption p = 0.087

## Discussion

Caregiver responses to children with severe and uncomplicated malaria vary. Overall, staying at home was the most common response to illness by caregivers, followed by care seeking at a drug shop, which was more common among caregivers of children with severe malaria. At every interval following illness onset, compared to caregivers of children with uncomplicated malaria, caregivers of those with severe malaria were more likely to have delayed seeking of appropriate care. This difference was most pronounced in the initial 24 h following fever onset. During this interval, care seeking at a drug shop as the initial response to illness was significantly associated with delay in seeking appropriate care.

Staying home is a common initial response to illness among caregivers of children with fever, accounting for 68% to 83% of initial caregiver responses to illness in three prior studies in sub-Saharan Africa [19–21]. At home, majority of caregivers provide self-medication [22], as was observed in our study, in which half of the children initially stayed home and were provided with medication by their caregivers. Upon adjustment, a significant association between staying at home on self-medication and delayed care-seeking was not found. However, the practice has the potential to contribute to delays in seeking appropriate care. In 2000, in the interest of broadening access to care, the Roll Back Malaria Summit in Abuja passed a declaration allowing for treatment of malaria at home [23]. Since then, and based on experimental studies that demonstrated benefit [24, 25], the WHO has advocated for Home Management of Malaria (HMM) as an intervention to increase prompt access to effective anti-malarials [26]. However, despite being associated with prompt initiation of treatment, inappropriate treatment at home compromises the effectiveness of this approach [27–29]. Integration of HMM into community case management of childhood illness (iCCM), an enhanced version of HMM, has also been associated with high levels of inappropriate treatment [30, 31]. Despite limitations, HMM and iCCM, if effectively implemented, offer home and community based initiatives for providing prompt treatment to populations at risk of malaria.

In this study, drug shops were the most common initial service provider sought, particularly among caregivers of children with severe malaria. Previous studies in Uganda indicated that more than half of caregivers (range 51.7–56.6%) used private outlets to treat childhood fevers [32]. Similar findings were reported in Ghana, where half of patients visiting a health facility with severe malaria had previously sought care at a drug shop [33]. Employed caregivers were more likely to seek care at drug shops, potentially attributed to their ability to afford these services or the advantages of physical accessibility or flexible opening hours.

However, despite their popularity drug shops have been associated with sub-optimal services for children suffering from malaria [34–36]. The results of this study linked drug shops with sub-optimal practices, as reported by caregivers, including low rates of physical examination of sick children, malaria diagnostic testing, and referral. These findings are consistent with reports from Tanzania indicating that children with confirmed malaria are less likely to receive an ACT from a drug shop as compared to a public facility [12]. In Uganda and Kenya, surveys of health facilities indicate that most drug shops stocked poor quality anti-malarials in addition to stocking non-recommended monotherapies [37, 38]. Low testing rates at drug shops indicate that most children were managed for malaria presumptively, thus not following WHO guidelines.

Due to their close proximity and popularity among rural populations [34, 39, 40], there is growing interest in utilizing drug shops to extend health care services [41, 42]. However, despite their potential to provide prompt treatment [43, 44], because treatment may be inappropriate, drug shops may actually delay access to appropriate care [45, 46], increasing the risk of progression of uncomplicated to severe malaria. Regrettably, the findings of this study point to this problem, evidenced by the observation that, despite seeking care promptly, caregivers who initially sought care at drug shops were likely to delay seeking appropriate care within the first 24 h of illness onset. This finding is substantiated by caregiver reports, which indicated that drug shops as compared to public health facilities were significantly less likely to refer patients to another facility. Indeed, a recent study conducted in Uganda suggested that providers at drug shops are not motivated to refer patients, presumably due to economic benefits to shopkeepers of providing care [14]. Surprisingly, compared to drug shops that frequently prescribed anti-malarials, prescription of anti-malarials by public health facilities was low, especially at public hospitals. This paradox may be explained by the fact that public health facilities were caring for children who had probably received treatment prior to presenting at the facility. Additionally, public facilities were more likely to refer patients’ unlike drug shops that did not. Despite limitations, by providing medicines promptly, drug shops provide vital services close to communities at risk. Indeed, through training and price subsidies [4, 47] the quality of services offered at drug shops has been improved [35]. However, it remains unclear if acceptable standards of managing children with malaria can be attained and gains sustained at drug shops [48–51].

This study had some limitations inherent to the case–control study design. First, cases were enrolling from a referral hospital, excluding cases that were not hospitalized at this facility, limiting the representativeness of the study population. For example, children who died at home from severe malaria were not represented. Second, recall by caregivers, including interventions by different facilities, could have been a source of bias influenced by the state of the child at the time of enrolment. Third, despite assuring respondents of confidentiality, caregivers may have been reluctant to report unconventional choices of care for fear of perceived rebuke. Fourth, the estimated population incidence of severe malaria used to adjust for biased representation among cases in the study population may have resulted in inaccurate population estimates, compromising validity of the study findings related to secondary outcomes. Lastly, recording of caregiver responses in a sequential manner limited the ability to study the influence of concurrent responses on outcomes.

## Conclusion

Findings from this study provide useful insights about caregiver responses to illness and the impact of these responses on time to seek appropriate care for children with malaria. Caregivers preferred to stay at home as the initial response to illness, and if they sought care outside the home, drug shops were preferred. Unfortunately, visits to drug shops led to delay in seeking appropriate care, probably due to in-appropriate care provided. Albeit limitations, drug shops provide an opportunity to bridge the gap and by promptly providing effective anti-malarial treatment to children with malaria, drug shops could provide benefit. However, for benefits to be realized and sustained, the role of drug shops in providing care to sick children needs to be re-defined, appropriate care protocols established, and effective mechanisms of regulating drug shops instituted. Finally, prioritizing and directing limited resources to holistically strengthening the existing public health system may be a more rational approach.

## Additional file

**Additional file 1.** Itinerary of events.
