# Supplementary figures and images for: Caregiver responses and association with delayed care-seeking in children with uncomplicated and severe malaria
Source: Malar J. 2018 Dec 18;17:476. doi: 10.1186/s12936-018-2630-9 (PMC6299589; doi:10.1186/s12936-018-2630-9)

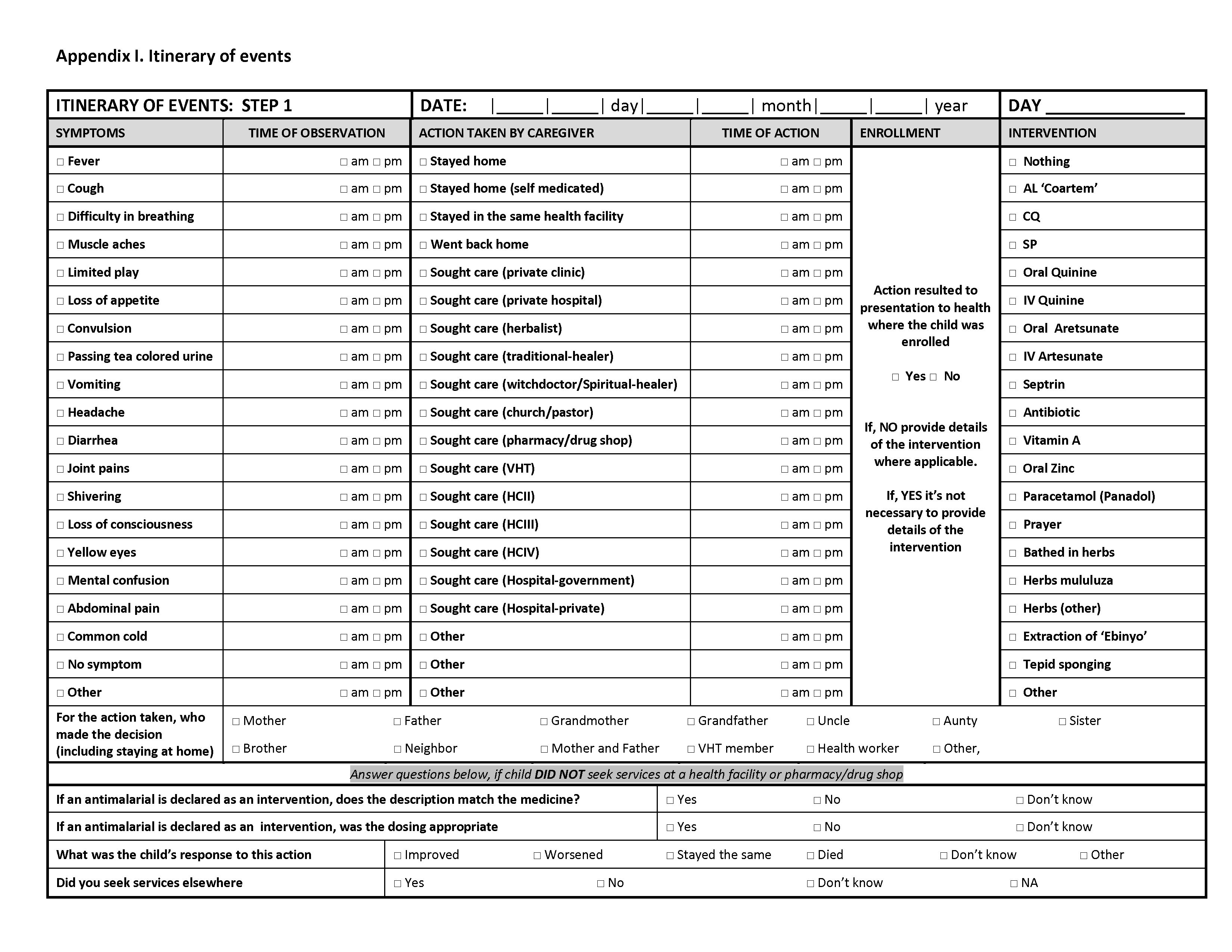

Supplement: Supplementary file 1 — Additional file 1. Itinerary of events. [file 12936_2018_2630_MOESM1_ESM.tif]
